# Supplementary material for: Multi-purpose cash transfers and health among vulnerable Syrian refugees in Jordan: A prospective cohort study
Source: PLOS Glob Public Health. 2022 Nov 2;2(11):e0001227. doi: 10.1371/journal.pgph.0001227 (PMC10021566; doi:10.1371/journal.pgph.0001227)
Supplement: S1 File — Additional details on sampling methods, including change in intervention receipt among study households during the study period and analyzed sample follow-up by intervention receipt. (PDF) [file pgph.0001227.s002.pdf]

SUPPLEMENTAL METHODS

Table 1: Change in Intervention Receipt among Study Households During the Study Period

	**Continue on Intervention**		**Continue as Control**		**Switch: Intervention to Control** [a]		**Switch: Control to Intervention**	
	N	%	N	%	N	%	N	%
Amman	123	34.1%	145	37.1%	32	38.6%	14	28.0%
Irbid	83	23.0%	89	22.8%	20	24.1%	14	28.0%
Mafraq	68	18.8%	63	16.1%	12	14.5%	14	28.0%
Zarqa	36	10.0%	37	9.5%	10	12.0%	5	10.0%
Rest of Jordan [b]	51	14.1%	57	14.6%	9	10.8%	3	6.0%
Total	**361**	**40.8%**	**391**	**44.2%**	**83**	**9.4%**	**50**	**5.6%**

[a] Excluded from final analysis
[b] Rest of Jordan includes Ajloun, Aqaba, Balqa, Jerash, Karak, Maan, Madaba, and Tafiela governorates

Table 2: Analyzed Sample Follow-Up by Intervention Receipt

	**Continue on Intervention** [a]		**Switch: Control to Intervention** [a]		**Continue as Control**		**Total Analyzed Sample**	
	N	%	N	%	N	%	N	%
Complete follow-up	361	95.5%	50	98.0%	391	87.3%	802	91.4%
Lost to follow-up	17	4.5%	1	2.0%	57	12.7%	75	8.6%
Refused endline interview	17	4.5%	0	0.0%	25	5.6%	42	4.8%
Ineligible	0	0.0%	0	0.0%	0	0.0%	0	0.0%
Unreachable	0	0.0%	1	2.0%	32	7.1%	33	3.8%
Total	**378**	**43.1%**	**51**	**5.8%**	**448**	**51.1%**	**877**	**100%**

[a] Included as MPC group for final analysis

Table 3. Baseline Demographic Characteristics and Living Conditions of Participants Lost to Follow-up

		MPC HHs (N=18)	Control HHs (N=57)
**Principal Applicant/Household Head Characteristics**			
Female sex		13 (72.2%)	16 (28.1%)
Age (mean years)		60.4 (13.5)	37.8 (12.6)
Highest level of education	None	9 (50.0%)	8 (14.0%)
	Primary school	5 (27.8%)	24 (42.1%)
	Preparatory school	1 (5.6%)	13 (22.8%)
	Secondary school	3 (16.7%)	12 (21.1%)
Marital status	Married	6 (33.3%)	42 (73.7%)
	Widowed	8 (44.4%)	5 (8.8%)
	Never married / Divorced	0 (0.0%)	7 (12.3%)
**Household Demographic Characteristics**			
Household size (mean)		4.4 (2.2)	4.6 (2.3)
Dependency ratio [a] (mean)		1.3 (0.6)	0.9 (0.8)
Multiple UNHCR registration cases (%)		10 (55.6%)	24 (42.1%)
Vulnerable members (%)			
	Child(ren) <5 yrs	7 (38.9%)	30 (52.6%)
	Child(ren) ≤ 17 yrs	12 (66.7%)	41 (71.9%)
	Older adult(s) (>60 yrs)	13 (72.2%)	15 (26.3%)
	Member with a chronic health condition	16 (88.9%)	33 (57.9%)
	Member w/ disability or that needs daily support	5 (27.8%)	13 (22.8%)
**Living Conditions**			
Residence type	Apartment or house	14 (77.8%)	53 (93.0%)
	Single room	1 (5.6%)	3 (5.3%)
	Temporary shelter [b]	2 (11.1%)	0 (0.0%)
	Other [c]	1 (5.6%)	1 (1.8%)
Residence arrangement	Rented	1 (5.6%)	0 (0.0%)
	Hosted for free / rent paid by NGO/charity	16 (88.9%)	54 (94.7%)
	Owned	0 (0.0%)	3 (5.3%)
Crowding (mean # people/sleeping room)		2.1 (1.5)	1.8 (0.9)

Presented as N (%) or mean (standard deviation).
[a] Number of dependents divided by number of working age adults
[b] includes tent, prefab unit, collective center
[c] includes unfinished building, construction site, factory, or warehouse

Table 4. Baseline Household Economy and Receipt of Humanitarian Assistance Among Participants Lost to Follow-up

		MPC HHs (N=18)	Control HHs (N=57)
**Household Income and Expenditures (past month; mean US$ [a])**			
Income (excluding humanitarian assistance)		239.7 (223.4)	386.8 (370.6)
Total expenditures		444.8 (263.6)	649.9 (665.3)
**Total Humanitarian Assistance (past month) [b]**			
**Any regular transfer (% of HHs)**		17 (94.4%)	50 (87.7%)
Amount received (mean US$ [a] per HH)		234.4 (164.1)	114.5 (85.7)
Amount received (mean US$ [a] per HH member)		58.9 (29.4)	27.3 (23.2)
**In-kind assistance (past 3 months)**		1 (5.6%)	7 (12.3%)
**WFP Food Assistance (past month)**			
Current WFP recipients		17 (94.4%)	50 (87.7%)
Amount received (mean US$ [a] per HH)		94.2 (54)	93.2 (64.6)
Amount received (mean US$ [a] per HH member)		22.4 (6.7)	20.3 (8.1)
Transfer modality	E-Voucher	13 (76.5%)	44 (88.0%)
	Choice	4 (23.5%)	6 (12.0%)
**Asset Sales and Borrowing**			
Sold assets in past 3 months (%)		0 (0.0%)	17 (29.8%)
Borrowed money in past 3 months (%)		11 (61.1%)	35 (61.4%)
Current debt	Any Debt	11 (78.6%)	42 (80.8%)
	Amount of debt (among those w/ debt; mean US$ [a])	333.9 (378.1)	570 (623)

Presented as N (%) or mean (SD).

[a] Exchange rate: 1 JOD = 1.41 US$; [b] includes UNHCR, WFP, and regular monthly assistance from other less common sources
