# Supplementary material for: Multi-purpose cash transfers and health among vulnerable Syrian refugees in Jordan: A prospective cohort study
Source: PLOS Glob Public Health. 2022 Nov 2;2(11):e0001227. doi: 10.1371/journal.pgph.0001227 (PMC10021566; doi:10.1371/journal.pgph.0001227)
Supplement: S2 File — The questionnaire developed and used for this study. (PDF) [file pgph.0001227.s003.pdf]

Multi-purpose cash transfers and health among vulnerable Syrian refugees in Jordan: Study Questionnaire

1. SECTION 1. INTERVIEW BACKGROUND INFORMATION
Date of Enrollment

2. Interviewer number

3. UNHCR Case Number from sampling list

4. Assigned sample group
Choose one response
- MPC
- Control

5. Household Study ID

6. Arabic consent script
7. Arabic consent script, continued

8. Do you agree to participate in this study?
Choose one response
- Yes, agree to participate
- No, refuse to participate   If this response, jump to 307
- Yes, agree to participate but asked to postpone interview If this response, jump to 307

9. SECTION 2: INFORMATION ABOUT RESPONDENT AND UNHCR CASE
Identify the Principal Applicant of the case number; if not present, an adult included in the same registration case that is considered capable and reliable for the interview may be interviewed. If possible, select the mother of the children or an adult who is engaged in decision making for the household.

10. Please tell me your UNHCR case number.
INTERVIEWER: Does the case number provided match the sampled case number?
If not, probe to find someone on the sampled case number. If someone on the sampled case number cannot be identified, select "no matching case number not identified" and end the interview.
Choose one response
- Yes, matching case number provided
- No, matching case number not identified If this response, jump to 307

11. Record UNHCR case number

12. Given Name of Participant

13. Family Surname(s)

14. Cell phone number 1

15. Relationship to phone owner
Choose one response
- Shared household phone
- Self/personal phone

- Another household member's personal phone
- Neighbor
- Other

16. Alternate cell phone number where participant can be reached

17. Relationship to alternate phone owner

Choose one response
- Shared household phone
- Self/personal phone
- Another household member's personal
- Neighbor
- Other

18. Respondent sex

Choose one response
- Male
- Female

19. How old are you? (in years)

20. What is the highest level of education you have completed?

Choose one response
- No formal schooling
- Some primary school
- Primary school completed
- Preparatory school completed
- Secondary school completed
- College/ University completed
- Don’t Know
- Refused

21. What is your marital status?

Choose one response
- Married
- Widowed
- Never married
- Divorced
- Refused

22. What is your relationship to the principal applicant on the UNHCR case?

Choose one response
- I am the principal applicant If this response, jump to 26
- Wife/Husband
- Mother/Father
- Daughter/Son
- Brother/Sister
- Father-in-law/Mother-in-law
- Son-in-law/daughter-in-law
- Brother-in-law/Sister-in-law
- Extended family (uncle/aunt/cousin/niece/nephew etc)
- No family relationship – guest

- Other

23. Is the principal applicant a male or a female?
Choose one response
- Male
- Female

24. How old is the principal applicant (in years)?

25. What is the highest level of education the principal applicant has completed?
Choose one response
- No formal schooling
- Some primary school
- Primary school completed
- Preparatory school completed
- Secondary school completed
- College/ University completed
- Don't Know
- Refused

26. Which governorate do you currently live in?
Choose one response
- Ajloun
- Al Aqabah
- Al Balqa
- Al Karak
- Al Mafraq
- Amman
- At Tafilah
- Az Zarqa
- Irbid
- Jarash
- Ma'an
- Madaba

27. Which district do you currently live in?
Choose one response
- Kufr Anja
- Qasabat Ajloun
- Al Quwayra
- Qasabat Al Aqabah
- Ash Shuna Al Janoubyah
- Ayn Al Basha
- Deir Alla
- Mahis Wa Al Fuhays
- Qasabat As Salt
- Al Aghwar Al Janoobiyah
- Al Mazar Al Janoubi
- Al Qasr
- Al Qatrana

- Ayy
- Fqou
- Qasabat Al Karak
- Al Badiah Al Shamaliyah
- Al Badiah Al Shamaliyah Al Gharbiyah
- Al Ruwaished
- Qasabat Al Mafraq
- Al Jamiah
- Al Jiza
- Al Quaysmh
- Marka
- Mwaqqar
- Naaur
- Qasabat Amman
- Sahab
- Wadi As Sir
- Al Hasa
- Busayrah
- Tafilah
- Al Hashimiya
- Ar Rusayfa
- Qasabat Al Zarqa
- Al Aghwar Al Shamalyah
- Al Koura
- Al Mazar Ash Shamali
- Al Wastiyya
- Ar Ramtha
- At Tayba
- Bani Kinana
- Bani Obayd
- Irbid
- Qasabat Jarash
- Al Husayniyya
- Al Petra
- Al Shobak
- Qasabat Maan
- Qasabat Madaba
- Theeban

28. Housing Type
Choose one response

- Apartment/house (finished) / شقة/ منزل - غير مشترك

- Single room in apartment/house / غرفة ضمن شقة أو منزل

- Single room in other location (addition to home, in a workplace location such as garage or farm) / غرفة مفردة في مكان آخر بالإضافة إلى المنزل، في مكان العمل مثل مرآب أو مزرعة-

- Unfinished building or construction site / مبنى غير مكتمل أو ورشة

- Factory or warehouse / مصنع او مستودع

- Tent or temporary shelter built from other materials / خيمة أو مأوى مؤقت مبني من مواد أخرى

- Prefab unit / باراكس

- Collective center/shelter / مأوى جماعي

- Other / أخر

29.طبيعة السكن
Occupancy Type
Choose one response
- Apartment/house (finished)
- Single room in apartment/house
- Single room in other location (addition to home, in a workplace location such as garage or farm)
- Unfinished building or construction site
- Factory or warehouse
- Tent or temporary shelter built from other materials
- Prefab unit
- Collective center/shelter
Other

29. Occupancy Type
Choose one response
- Owned If this response, jump to 31
- Rented (direct rent payment) If this response, jump to 31
- Rented (in exchange of work) If this response, jump to 31
- Hosted for free If this response, jump to 31
- Rented/paid for by NGO/charity If this response, jump to 31
- Squatting If this response, jump to 31
- Other (specify)

30. Provide description for "other"

31. Excluding bathrooms/toilets, how many rooms does your residence have?

32. SECTION 3: HOUSEHOLD DEMOGRAPHICS
READ ALOUD: Now I will ask you some questions about your household. A household includes people who share a living space AND share meals and financial resources.
INTERVIEWER: Probe to ensure the respondent is correctly identifying the household. Households may include more than one UNHCR registration case. Extended family members that share the living space should only be considered household members if they regularly share meals and financial resources. Household members may or may not be biologically related. Short term visitors who stay for less than one month should not be counted household members.

33. How many people currently live in this household?

34. How many households live together in this residence?

35. How many people in the household are less than 5 years of age?

36. How many people in the household are age 5 to 17 (including age 17)?

37. How many people in the household are age 18 to 59 (including age 59)?

38. How many people in the household are age 60 or higher?

39. How many household members have chronic health conditions?
Chronic health conditions are deteriorations in health which require regular visits to a doctor or regular medication or treatment. For example, hypertension, kidney disease, neurological conditions like stroke or migraines, arthritis, cancer, or mental illness. Some people may have more than one chronic condition, but should be counted once.

40. How many household members have a disability or special needs (physical, hearing, visual, or mental disability)?
These are long term conditions which are not likely to change greatly in the next few months and limit normal function of a household member.

41. How many household members are in need of support to do daily activities due to other conditions (such as frailty or limitations due to old age)?

42. How many UNHCR registration cases are in this household?
A registration case is defined as a group of people that share the same UNHCR registration number (and are listed together on the same registration certificate or were recorded together when you received your token).

43.Please enter the first additional UNHCR case number in the household

44.Please enter the second additional UNHCR case number in the household

45. SECTION 4: HOUSEHOLD ECONOMY
READ ALOUD: Please estimate your household's expenses in the last month for the following expense categories (in JD; enter '999' for not disclosed or '0' if household had no expense)
INTERVIEWER: if the household has multiple registration cases, remind the respondent to report this information for the entire household (those that live together and share financial resources/meals).

46. Housing / rent

47. Electricity or generator

48. Gas / Fuel / Wood (for cooking and heating only)

49. Water

50. Food

51. Consumable household items (toilet paper, soap, etc. Does not include food or larger purchases such as furniture)

52. Combined: Food and household items
NOTE: If respondent is unable to separate food and other household item expenditures, enter combined amount. If separate expenditures entered for each category, enter 0.

53. Telecommunication, internet, mobile subscription

54. Transportation

55. Health care (consultation fees, diagnostic testing, medicines)

56. Education (include transportation and supplies)

57. Clothes

58. Payment of debt

59. Any other large expenses that you have not mentioned? (Enter the amount of other large expenses in the last month)

60. Specify other large expenses

61. Were your household's expenditures in the past month similar to your usual expenditure levels?
Choose one response
- Yes   If this response, jump to 63
- No, expenditures were higher than usual
- No, expenditures were lower than usual
- Don't Know   If this response, jump to 63

62. If lower or higher, please explain:

63. Now I would like to ask a few questions about your households' spending related to health in the last month. For each question, please exclude spending using vouchers for health.
In the past month, has your household spent money on health (including medical consultations, diagnostic tests, medications, and/or transportation to receive these)?
Choose one response
- Yes
- No   If this response, jump to 73
- Don't Know   If this response, jump to 73

64. In the past month, how much has your household spent on medical consultations (in JD)? Do not include the cost of medicines.
If the answer is "I don't know", please insert number 999.
If there were no expenses, enter "0"

65. In the past month, how much has your household spent on medical diagnostic tests (in JD)?
If the answer is "I don't know", please insert number 999.
If there were no expenses, enter 0

66. In the past month, how much has your household spent on medications (in JD)?
If the answer is "I don't know", please insert number 999.
If there were no expenses, enter 0

67. In the past month, how much has your household spent on transportation to receive medical care, diagnostic tests, or prescribed medication (in JD)?
If the answer is "I don't know", please insert number 999.
If there were no expenses, enter 0

68. Were your household's expenditures on health in the past month similar to your usual expenditure levels?
Choose one response

- Yes   If this response, jump to 71
- No, expenditures were higher than usual
- No, expenditures were lower than usual
- Don’t Know   If this response, jump to 71

69. If lower or higher, please explain:

70. How much does your household spend on health in a usual month (including medical consultations, diagnostic tests, medications, and/or transportation to receive these)?
Enter ‘999’ for don't know

71. In the past month, did any organization give you money, either before or after the health expenses were incurred, that was intended to help cover medical costs?
Choose one response
- Yes
- No   If this response, jump to 73
- Don’t Know   If this response, jump to 73

72. How much money did they provide (in JD)?

73. Please estimate your household's total income in the last month (in JD). This may include income from a variety of sources such as salaries, pensions, investments or remittances. Do not include cash or vouchers received as humanitarian assistance.

74. How many household members worked in the last month?

75. From which type of employment is the households’ largest income source?
Choose one response
- Agriculture
- Construction
- Manufacturing
- Hotel/restaurant/sales
- Domestic service job
- Professional/salaried position
- Self-employment
- Transportation (eg: taxi driver)
- Doorman / building keeper
- Other

76. How much income came from this source in the last month (in JD)?

77. Is this full-time or part-time employment?
Choose one response
- Full time
- Part time

78. Is this income regular?
Choose one response
- Regular (consistent/stable income)
- Irregular (varies daily/weekly)
- Seasonal (varies monthly/seasonally)

79. Did the household have employment income from any other sources?
Choose one response
- Yes
- No   If this response, jump to 86

80. From which type of employment is the households' second largest income source?
Choose one response
- Agriculture
- Construction
- Manufacturing
- Hotel/restaurant/sales
- Domestic service job
- Professional/salaried position
- Self-employment
- Transportation (eg: taxi driver)
- Doorman / building keeper
- Other

81. How much income came from this source in the last month (in JD)?

82. Is this full-time or part-time employment?
Choose one response
- Full time
- Part time

83. Is this income regular?
Choose one response
- Regular (consistent/stable income)
- Irregular (varies daily/weekly)
- Seasonal (varies monthly/seasonally)

84. Did the household have employment income from any other sources?
Choose one response
- Yes
- No   If this response, jump to 86

85. From which other types of employment did the household earn income?
Choose all that apply
- Agriculture
- Construction
- Manufacturing
- Hotel/restaurant/sales
- Domestic service job
- Professional/salaried position
- Self-employment
- Transportation (eg: taxi driver)
- Doorman / building keeper
- Other

86. In the last month, did your household obtain income from any of the following sources? If yes, enter monthly amount in JD (total). If no, enter "0"

87. Profit from rental property you own (in Syria or elsewhere)

88. Remittances from family (abroad or employed elsewhere)

89. Gift from family/friend/other person

90. Loan

91. Asset Sales

92. Other
Enter the amount of income received from other sources.

93. Specify other income

94. SECTION 5: RECEIPT OF HUMANITARIAN ASSISTANCE

95. Since arriving to Jordan, have you received regular monthly cash transfers from UNHCR? This is a regular transfer of the same amount provided every month to help households meet basic needs. Do not include intermittent transfers such as those provided in only in winter.
If yes, for how many years?
INTERVIEWER: If the household has come off and on UNHCR cash transfers, ask them to estimate each time period separately and then combine them to find the total length of time receiving UNHCR cash transfers. Only include cash transfers from UNHCR.
Choose one response
- Never received UNHCR assistance If this response, jump to 115
- < 6 months
- 6 months to < 1 year
- 1 year to < 2 years
- 2 years to < 3 years
- 3 or more years

96. Does your household currently receive monthly cash assistance from UNHCR?
Choose one response
- Yes
- No If this response, jump to 115
- Don't Know If this response, jump to 115

97. If yes, which case registration number(s) receive monthly cash assistance from UNHCR?
Enter case registration numbers, separating each with a comma (i.e. case#1,case#2,case#3....)

98. How much money did your household receive from UNHCR last month (in JD)?

99. READ ALOUD: Now I would like to ask you about the use of UNHCR cash transfers. For each category that I read, please tell if you spend UNHCR cash on it or not. If yes, please tell me the expenditure amount for the last month. (in JD; Record 0 for none, 999 for don't know)

100. Housing / rent

101. Electricity or generator

102. Gas / Fuel / Wood (for cooking and heating only)

103. Water

104. Consumable household items (toilet paper, soap, etc. Does not include larger purchases such as furniture)

105. Telecommunication, internet, mobile subscription

106. Food

107. Transportation

108. Medicines

109. Health care (consultation fees, diagnostic testing)

110. Education (include transportation and supplies)

111. Clothes

112. Payment of debt

113. Anything else?
Enter the amount of UNHCR cash transfers spent on other items.

114. Specify other cash spending

115. Since arriving to Jordan, have you received regular monthly assistance from the World Food Programme?
If yes, for how many years?
INTERVIEWER: If the household has come off and on WFP assistance, ask them to estimate each time period separately and then combine them to find the total length of time receiving WFP assistance. Only include assistance from WFP.
Choose one response
- Never received If this response, jump to 118
- < 6 months
- 6 months to < 1 year
- 1 year to < 2 years
- 2 years to < 3 years
- 3 or more years

116. Does your household currently receive monthly cash or voucher assistance from the World Food Programme?
Choose one response
- Yes - e-voucher redeemable in stores
- Yes - choice of ATM or store redemption
- No If this response, jump to 118
- Don't Know If this response, jump to 118

117. How much money did your household receive from WFP last month (in JD)?

118. Since arriving to Jordan, have you received regular monthly cash or voucher assistance from any other organization?

Include only cash transfers that are provided on a monthly basis for the same amount.
If yes, for how many years? If multiple sources, record the longest.
INTERVIEWER: If the household has come off and on other cash/voucher assistance, ask them to estimate each time period separately and then combine them to find the total length of time receiving other cash/voucher assistance.
Choose one response
- Never received   If this response, jump to 123
- < 6 months
- 6 months to < 1 year
- 1 year to < 2 years
- 2 years to < 3 years
- 3 or more years

119. Does your household currently receive regular monthly cash or voucher assistance from any other organization?
Include only cash transfers that are provided on a monthly basis for the same amount.
Choose one response
- Yes
- No   If this response, jump to 123
- Don't Know   If this response, jump to 123

120. Which organization(s) provides assistance?

121. Is the assistance unrestricted or is it intended to be used for certain types of expenses only?
Include only transfers that are provided on a monthly basis for the same amount.
Choose all that apply
- Unrestricted cash
- Cash for rent
- Voucher for rent
- Cash for education
- Cash for shelter
- Cash for health
- Other cash (specify)

122. In total, how much did your household receive in cash/voucher assistance last month from all sources other than UNHCR and WFP (JD)?

123. Has your household received any other type of cash or voucher assistance in the past 3 months?
Include any cash/voucher assistance that was received from humanitarian organizations on an irregular basis (most often a single transfer). For example, cash for child birth or winterization.
Choose one response
- Yes
- No If this response, jump to 127
- Don't Know   If this response, jump to 127

124. From which organization(s)?

125. How much cash/voucher assistance was received, in total, from all organizations, in the past 3 months (in JD)?
Enter the combine amount from all sources. If necessary, help the respondent by listing transfers from each source separately; then record the total.

126. What was the assistance provided for?

127. Did your household receive any in-kind assistance in the past 3 months?
Choose one response
- Yes
- No If this response, jump to 130
- Don't Know   If this response, jump to 130

128. What type(s) of assistance were received?
Choose all that apply
- Accommodation
- Shelter materials/repairs
- Utilities (fuel, electricity, water)
- Household items / Clothing
- Food
- Medication
- Health services
- Education
- Business/livelihood inputs
- Other (specify)

129. Provide description for "other". Leave blank if "other" was not selected in previous question.

130. SECTION 6: ASSET SALES AND BORROWING

131. In the past 3 months, have you had to sell any assets in order to pay for routine household expenses?
Choose one response
- Yes
- No If this response, jump to 138
- Don't Know   If this response, jump to 138

132. What types of assets did you sell?
Choose all that apply
- Jewelry or gold
- Household assets (appliances, electronics, etc.)
- Vehicle (car, motorcycle)
- Property (land, house)
- Productive assets (personal or business items used for income generation)
- Other

133. How much money did your household earn from asset sales in the past 3 months (in JD)?

134. What did you use the majority of money from asset sales for?
If money was spent on multiple categories, record the category where most of the money was spent.
Choose one response
- Rent   If this response, jump to 136
- Food   If this response, jump to 136
- Medication   If this response, jump to 136
- Health service   If this response, jump to 136
- Education   If this response, jump to 136
- Business/livelihood   If this response, jump to 136
- Shelter repairs   If this response, jump to 136
- Utilities (fuel, electricity, water)   If this response, jump to 136
- Household items / Clothing   If this response, jump to 136

- Other (specify)

135. Provide description for "other"

136. Did you spend money from asset sales on anything else?
Choose all that apply
- No
- Rent
- Food
- Medication
- Health service
- Education
- Business/livelihood
- Shelter repairs
- Utilities (fuel, electricity, water)
- Household items / Clothing
- Other (specify)

137. Provide description for "other". Leave blank if "other" was not selected in previous question.

138. In the past 3 months, have you had to borrow any money in order to pay for routine household expenses?
Choose one response
- Yes
- No   If this response, jump to 145
- Don't Know   If this response, jump to 145

139. From whom have you borrowed? (Note: if borrowing from more than one source, select the source from which the most was borrowed)
Choose all that apply
- Family
- Friends
- Informal lender
- Microfinance organization
- Bank
- Shawish
- Store (bought food on credit)
- Local associations/charity (for example, a mosque)
- Landlord
- Stranger
- Other

140. How much money did your household borrow in the past 3 months (in JD)?
If necessary, help the respondent by listing the amount borrowed from each source separately; then record the total.

141. What did you use the majority of borrowed money for?
If money was spent on multiple categories, record the category where most of the money was spent.
Choose one response
- Rent   If this response, jump to 143
- Food   If this response, jump to 143
- Medication   If this response, jump to 143
- Health service   If this response, jump to 143

- Education   If this response, jump to 143
- Business/livelihood   If this response, jump to 143
- Shelter repairs   If this response, jump to 143
- Utilities (fuel, electricity, water)   If this response, jump to 143
- Household items / Clothing   If this response, jump to 143
- Other (specify)

142. Provide description for "other"

143. Did you spend borrowed money on anything else?
Choose all that apply
Choose all that apply
- No
- Rent
- Food
- Medication
- Health service
- Education
- Business/livelihood
- Shelter repairs
- Utilities (fuel, electricity, water)
- Household items / Clothing
- Other (specify)

144. Provide description for "other". Leave blank if "other" was not selected in previous question.

145. How much money is currently owed by your household (in JD)? Include money borrowed within the past 3 months in addition to any other outstanding debt.

146. SECTION 7: CHILD HEALTH CARE SEEKING
Read aloud: Now I would like to ask some questions about the last time a child (under 18 years old) in your household needed health care.

147. DO NOT READ ALOUD: Is there anyone under 18 years of age in this household?
Choose one response
- Yes
- No   If this response, jump to 215

148. When was the last time a CHILD (under 18 years old) in your household had a health problem that you felt needed medical care?   Only include health problems that occurred while in Jordan, even if you did not take the child to a doctor, pharmacy or other medical provider.
INTERVIEWER: If more than two children were sick at the same time as the caregiver which child was the sickest and answer the following set of questions only for the sickest child.
Choose one response
- Less than two weeks ago
- Between 2 weeks and less than 1 month ago
- Between 1 month and less than 3 months ago
- Between 3 months and less than 6 months ago
- Between 6 months and less than 1 year ago
- More than 1 year ago   If this response, jump to 215
- Never needed care in Jordan   If this response, jump to 215

- Don't know If this response, jump to 216

149. How old is this child currently?
NOTE: record age in years. If the child is 0-11 months old, you should write "0". If the child is 12-23 months old, you should write "1". If the child is 24-35 months old, you should write "2". etc.

150. Child sex
Choose one response
- Male
- Female

151. Which reason best describes why health care was needed? (if more than one, record the most concerning)
Choose one response
- Fever If this response, jump to 153
- Diarrhea If this response, jump to 153
- Respiratory problem such as cough, cold, flu or difficulty breathing (not chronic) If this response, jump to 153
- Asthma If this response, jump to 153
- Injury If this response, jump to 153
- Dental care If this response, jump to 153
- Behavioral or emotional problem If this response, jump to 153
- Eye problem If this response, jump to 153
- Ear problem If this response, jump to 153
- Skin problem If this response, jump to 153
- Worms If this response, jump to 153
- Other (specify)

152. Provide description for "other"

153. Did the child receive medical attention for the condition?
Choose one response
- Yes, sought and received
- No, did not seek care If this response, jump to 213
- No, sought but no care received If this response, jump to 213
- Don’t Know If this response, jump to 215

154. Where did (child's name) first seek care for the condition?
Choose one response
- Government primary health care center
- Government comprehensive care center
- Private Jordanian clinic or doctor
- Islamic charity (free or low cost services to Syrian refugees)
- NGO or other non-religious charity (free or low cost services to Syrian refugees)
- Hospital
- Syrian doctor or clinic
- Pharmacy
- Shop or Other

155. What was the reason for choosing to seek care from that provider? (Record primary reason)
Choose one response
- Free consultation services
- Low cost consultation services
- Closest to place of residence

- No other facility nearby
- Like staff/quality/family doctor
- Free or reduced cost medications provided
- Free or reduced diagnostic tests provided
- Availability of needed services
- Medical emergency
- Short waiting time / easy to get an appointment
- Not sick enough to take to health facility
- Did not know any other providers
- Other (specify)

156. Provide description for other. Leave blank if other was not selected in the previous question.

157. What type of hospital was used?
Choose one response
- Private hospital

- Public hospital

- NGO

- Don't know

158. What type of visit was this?
Choose one response
- Emergency room visit (where people receive urgent care when they are very ill) If this response, jump to 165
- Outpatient visit If this response, jump to 165
- Inpatient admission (when you are given a bed at the hospital and stay overnight or longer)

159. How many nights was the hospital stay?

160. Did UNHCR or any other organization assist in paying for some of the fees?
Choose one response
- Yes, UNHCR
- Yes, other organization
- Yes, both
- No, none
- Don't Know

161. Did you/your household have to pay for any of the cost yourself?
Choose one response
- Yes
- No If this response, jump to 165
- Don't Know If this response, jump to 165

162. How much did your household pay for the hospital visit (in JD, excluding the amount paid by any organization on your behalf)?

163. Did you pay for the hospital visit on credit?
Choose one response

- Yes
- No If this response, jump to 165

164. How much is still owed (in JD)?

165. Were any medicines prescribed at this visit?
Choose one response
- Yes, provided at the health facility If this response, jump to 167
- Yes, but purchased at another location
- Yes, medicine prescribed but not obtained
- No medicine prescribed If this response, jump to 167

166. Why were you not able to get the medicines that were prescribed? (Record the primary reason)
Choose one response
- Medication was out of stock at the facility
- Medication was not available at the facility
- Medication was too expensive
- Other

167. Did your household have to pay for the visit?
Payment may include consultation fees for the provider, diagnostic testing, medicines, or transportation to get to/from the facility.
Choose one response
- Yes
- No If this response, jump to 175
- Don't Know If this response, jump to 175

168. What was the total amount that your household paid at the facility (JD)?

169. How much money did your household pay to the provider for consultation fees (JD)?

170. How much money did your household pay for diagnostic testing (JD)?

171. How much money did your household pay for medicines (at the facility, do not include medicines purchased elsewhere; in JD)?

172. Of the medicines you obtained at the health facility, did you get any on credit?
Choose one response
- Yes
- No If this response, jump to 174

173. How much is still owed (in JD)?

174. How much money did your household pay for transportation to/from the facility (in JD)?

175. Were there any diagnostic tests, treatments, or medicines that were recommended but that you could not afford?
Choose one response
- Yes
- No If this response, jump to 178

176.ما الذي طلب إجرائه ولم تستطيع تحمل كلفته المادية؟
What was recommended that you could not afford?
Choose all that apply
- Diagnostic tests
- Medication
- Specialist Consultation
- Medical Equipment
- Other

177. What was the total cost of recommended tests/treatments/medicines that were not obtained because of cost (in JD)?

178. Was (name) referred to another facility or provider for care?
Choose one response
- Yes, received referral care
- Yes, but did not receive referral care
- No If this response, jump to 202
- Don't Know If this response, jump to 202

179. What was (name) referred for?
Record primary reason if multiple reasons are provided.
Choose one response
- Diagnostic or laboratory test
- Specialist consultation
- Curative intervention (specific medication, surgery, procedure)
- Other

180. Where was the child referred?
Choose one response
- Government primary health care center If this response, jump to 182
- Government comprehensive care center If this response, jump to 182
- Private Jordanian clinic or doctor If this response, jump to 182
- Islamic charity (free or low cost services to Syrian refugees) If this response, jump to 182
- NGO or other non-religious charity (free or low cost services to Syrian refugees) If this response, jump to 182
- Syrian doctor or clinic If this response, jump to 182
- Hospital
- Other If this response, jump to 182

181. What type of hospital was used?
Choose one response
- Private hospital
- Public hospital
- NGO
- Don't know

182. What type of visit was this?
Choose one response
- Emergency room visit (where people receive urgent care when they are very ill) If this response, jump to 189
- Outpatient visit If this response, jump to 189
- Inpatient admission (when you are given a bed at the hospital and stay overnight or longer)

183. How many nights was the hospital stay?

184. Did UNHCR or any other organization assist in paying for some of the fees?
Choose one response
- Yes, UNHCR
- Yes, other organization
- Yes, both
- No, none
- Don't Know

185. Did you/your household have to pay for any of the cost yourself?
Choose one response
- Yes
- No If this response, jump to 189
- Don't Know If this response, jump to 189

186. How much did your household pay for the hospital visit (in JD, excluding the amount paid by any organization on your behalf)?

187. Did you pay for the hospital visit on credit?
Choose one response
- Yes
- No If this response, jump to 189

188. How much is still owed (in JD)?

189. Were any medicines prescribed at the referral visit?
Choose one response
- Yes, provided at the health facility If this response, jump to 191
- Yes, but purchased at another location
- Yes, medicine prescribed but not obtained
- No medicine prescribed If this response, jump to 191

190. Why were you not able to get the medicines that were prescribed? (Record the primary reason)
Choose one response
- Medication was out of stock at the referral facility
- Medication was not available at the referral facility
- Medication was too expensive
- Other

191. Did your household have to pay for the referral provider visit?
Payment may include consultation fees for the provider, diagnostic testing, medicines, or transportation to get to/from the facility.
Choose one response
- Yes
- No If this response, jump to 199
- Don't Know If this response, jump to 199

192. What was the total amount that your household paid at the referral facility (JD)?

193. How much money did your household pay to the referral provider for consultation fees (JD)?

194. How much money did your household pay at the referral facility for diagnostic testing (JD)?

195. How much money did your household pay for medicines at the referral facility (do not include medicines purchased elsewhere; in JD)?

196. Of the medicines you obtained at the referral facility, did you get any on credit?
Choose one response
- Yes
- No If this response, jump to 198

197. How much is still owed (in JD)?

198. How much money did your household pay for transportation to/from the referral facility (in JD)?

199. Were there any diagnostic tests, treatments, or medicines that were recommended at the referral facility but that you could not afford?
Choose one response
- Yes
- No If this response, jump to 202

200.ما الذي طلب إجرائه ولم تستطيع تحمل كلفته المادية؟
What was recommended that you could not afford?
Choose all that apply
- Diagnostic tests
- Medication
- Specialist Consultation
- Medical Equipment
- Other

201. What was the total cost of recommended tests/treatments/medicines that were not obtained because of cost (in JD)?

202. Were any medications prescribed for the child's illness that were not obtained at the facilities where you received care?
Choose one response
- Yes
- No If this response, jump to 215

203. Did you attempt to obtain these medications elsewhere?
Choose one response
- Yes
- No If this response, jump to 207

204. Where did you attempt to obtain the prescribed medications?
Choose all that apply
- At another facility (different from where the doctor was seen)
- Private pharmacy
- Syria
- Via a local contact/fixer
- Other

205. Provide description for "other". Leave blank if "other" was not selected in previous question.

206. Were you able to get all of the medications that were prescribed?
Choose one response
- Yes, all of the medications   If this response, jump to 209
- Yes, some of the medications
- No
- Don't Know   If this response, jump to 215

207. Why were you not able to get all of the medicines that were prescribed? (Record the primary reason)
Choose one response
- Household could not afford the medication
- Did not know where to get the medication
- Medication was out of stock at the facility
- Medication was out of stock at private pharmacy
- Symptoms improved/child began to feel better
- Household chose a different treatment
- Household decided the medicines were not needed
- Purchasing medication was not a priority
- Too far to travel to obtain medication
- Other (specify)

208. Provide description for "other". Leave blank if "other" was not selected in previous question.

209. Did your household have to pay for the medications received outside the facilities where you received care?
Choose one response
- Yes
- No If this response, jump to 215

210. How much money did your household pay for medicines outside the facility where you received care (do not include medicines purchased at the care facility; in JD)?

211. Of the medicines you obtained outside the health facilities where care was received, did you get any on credit?
Choose one response
- Yes
- No If this response, jump to 215

212. How much is still owed (in JD)?

213. What was the main reason for not seeking medical care for the child's health condition? (Record one, Primary reason)
Choose one response
- Could not afford provider costs   If this response, jump to 215
- Distance or lack of transportation   If this response, jump to 215
- Could not afford transportation costs   If this response, jump to 215
- Provider's equipment or drugs are inadequate   If this response, jump to 215
- Disliked previous visit(s) (attitude)   If this response, jump to 215
- Disliked previous visit(s) (long waiting time)   If this response, jump to 215
- Disliked previous visit(s) (quality of care)   If this response, jump to 215
- Could not take time off work or had other commitments   If this response, jump to 215
- Didn't know where to go   If this response, jump to 215
- Family decided care should not be sought (no male permission)   If this response, jump to 215

- Security concerns and/or check points   If this response, jump to 215
- Appointment scheduled / still waiting   If this response, jump to 215
- Other (specify)

214. Provide description for "other"

215. SECTION 8: HEALTH CARE SEEKING FOR CHRONIC CONDITIONS
Now I am going to ask some questions about chronic health conditions in your household. Chronic health conditions are those which last for long periods of time, often many years, and may never be cured. Examples of chronic conditions include hypertension or high blood pressure, diabetes, heart problems and long term respiratory diseases, although there are many others. Include only adults 18 years and older.

216. How many of your household members have been diagnosed with a chronic health condition such as diabetes or hypertension?

217. I would like to ask some questions about each person in your household that has been diagnosed with a chronic health condition.
Subform name : H3SD_R2HC_MPC_Enrollment_Jordan_NCDSub_v4
Subform keyword : NCD

218. SECTION 9: ACUTE ILLNESS
Read aloud: Now I would like to ask some questions about the last time an adult (over 18 years old) in your household needed health care for an acute illness.

219. When was the last time an adult (over 18 years old) in your household been acutely ill with a health problem where they needed medical care? Include health problems that occurred, even if the individual did not receive medical care from a doctor, pharmacy, or other medical provider. Do not include chronic medical conditions. INTERVIEWER: If more than two acute illness occurred within the past month, ask the respondent which was most severe and answer questions only for that occurrence.
Choose one response
- Less than two weeks ago
- Between 2 weeks and less than 1 month ago
- Between 1 month and less than 3 months ago
- Between 3 months and less than 6 months ago
- Between 6 months and less than 1 year ago
- More than 1 year ago   If this response, jump to 286
- Never needed care in Jordan   If this response, jump to 286
- Don't know If this response, jump to 286

220. How old is (name) currently (in years)?

221. (Name)'s sex
Choose one response
- Male
- Female

222. Which reason best describes why health care was needed? (if more than one, record the most concerning)
Choose one response
- Injury   If this response, jump to 224
- Infection or communicable disease (including cough, cold, flu, and diarrhoea) If this response, jump to 224
- Dental Care   If this response, jump to 224
- Skin problem   If this response, jump to 224

- Emotional or mental health If this response, jump to 224
- Complications related to pregnancy If this response, jump to 224
- Gynecological problem If this response, jump to 224
- Asthma/breathing difficulty   If this response, jump to 224
- Other (specify)

223. Provide description for "other"

224. Was medical attention received for the condition?

Choose one response
- Yes, sought and received
- No, did not seek care If this response, jump to 284
- No, sought but no care received   If this response, jump to 284
- Don't Know   If this response, jump to 286

225. Where did (name) first seek care for the condition?

Choose one response
- Government primary health care center
- Government comprehensive care center
- Private Jordanian clinic or doctor
- Islamic charity (free or low cost services to Syrian refugees)
- NGO or other non-religious charity (free or low cost services to Syrian refugees)
- Hospital
- Syrian doctor or clinic
- Pharmacy
- Shop or Other

226. What was the reason for choosing to seek care from that provider? (Record primary reason)

Choose one response
- Free consultation services
- Low cost consultation services
- Closest to place of residence
- No other facility nearby
- Like staff/quality/family doctor
- Free or reduced cost medications provided
- Free or reduced diagnostic tests provided
- Availability of needed services
- Medical emergency
- Short waiting time / easy to get an appointment
- Not sick enough to take to health facility
- Did not know any other providers
- Other (specify)

227. Provide description for other. Leave blank if other was not selected in the previous question.

228. What type of hospital was used?

Choose one response
- Private hospital
- Public hospital
- NGO
- Don't know

229. What type of visit was this?
Choose one response
- Emergency room visit (where people receive urgent care when they are very ill)   If this response, jump to 236
- Outpatient visit   If this response, jump to 236
- Inpatient admission (when you are given a bed at the hospital and stay overnight or longer)

230. How many nights was the hospital stay?

231. Did UNHCR or any other organization assist in paying for some of the fees?
Choose one response
- Yes, UNHCR
- Yes, other organization
- Yes, both
- No, none
- Don't Know

232. Did you/your household have to pay for any of the cost yourself?
Choose one response
- Yes
- No   If this response, jump to 236
- Don't Know   If this response, jump to 236

233. How much did your household pay for the hospital visit (in JD, excluding the amount paid by any organization on your behalf)?
234. Did you pay for the hospital visit on credit?
Choose one response
- Yes
- No   If this response, jump to 236

235. How much is still owed (in JD)?

236. Were any medicines prescribed at this visit?
Choose one response
- Yes, provided at the health facility   If this response, jump to 238
- Yes, but purchased at another location
- Yes, medicine prescribed but not obtained
- No medicine prescribed   If this response, jump to 238

237. Why were you not able to get all of the prescribed medicines at the health facility? (Record the primary reason)
Choose one response
- Medication was out of stock at the facility
- Medication was not available at the facility
- Medication was too expensive
- Other

238. Did your household have to pay for the visit?
Payment may include consultation fees for the provider, diagnostic testing, medicines, or transportation to get to/from the facility.
Choose one response
- Yes
- No If this response, jump to 246
- Don't Know   If this response, jump to 246

239. What was the total amount that your household paid at the facility (JD)?

240. How much money did your household pay to the provider for consultation fees (JD)?

241. How much money did your household pay for diagnostic testing (JD)?

242. How much money did your household pay for medicines (at the facility, do not include medicines purchased elsewhere; in JD)?

243. Of the medicines you obtained at the health facility, did you get any on credit?
Choose one response
- Yes
- No If this response, jump to 245

244. How much is still owed (in JD)?

245. How much money did your household pay for transportation to/from the facility (in JD)?

246. Were there any diagnostic tests, treatments, or medicines that were recommended but that you could not afford?
Choose one response
- Yes
- No If this response, jump to 249

247. What was recommended that you could not afford?
Choose all that apply
- Diagnostic tests
- Medication
- Specialist Consultation
- Medical Equipment
- Other

248. What was the total cost of recommended tests/treatments/medicines that were not obtained because of cost (in JD)?

249. Was (name) referred to another facility or provider for care?
Choose one response
- Yes, received referral care
- Yes, but did not receive referral care
- No If this response, jump to 273
- Don't Know If this response, jump to 273

250. What was (name) referred for?
Record primary reason if multiple reasons are provided.
Choose one response
- Diagnostic or laboratory test
- Specialist consultation
- Curative intervention (specific medication, surgery, procedure)
- Other

251. Where was (name) referred?

Choose one response
- Government primary health care center   If this response, jump to 253
- Government comprehensive care center   If this response, jump to 253
- Private Jordanian clinic or doctor If this response, jump to 253
- Islamic charity (free or low cost services to Syrian refugees)   If this response, jump to 253
- NGO or other non-religious charity (free or low cost services to Syrian refugees)   If this response, jump to 253
- Syrian doctor or clinic   If this response, jump to 253
- Hospital
- Other   If this response, jump to 253

252. What type of hospital was used?
Choose one response
- Private hospital
- Public hospital
- NGO
- Don't know

253. What type of visit was this?
Choose one response
- Emergency room visit (where people receive urgent care when they are very ill)   If this response, jump to 260
- Outpatient visit   If this response, jump to 260
- Inpatient admission (when you are given a bed at the hospital and stay overnight or longer)

254. How many nights was the hospital stay?

255. Did UNHCR or any other organization assist in paying for some of the fees?
Choose one response
- Yes, UNHCR
- Yes, other organization
- Yes, both
- No, none
- Don't Know

256. Did you/your household have to pay for any of the cost yourself?
Choose one response
- Yes
- No   If this response, jump to 260
- Don't Know   If this response, jump to 260

257. How much did your household pay for the hospital visit (in JD, excluding the amount paid by any organization on your behalf)?

258. Did you pay for the hospital visit on credit?
Choose one response
- Yes
- No   If this response, jump to 260

259. How much is still owed (in JD)?

260. Were any medicines prescribed at the referral visit?
Choose one response
- Yes, provided at the health facility   If this response, jump to 262

- Yes, but purchased at another location
- Yes, medicine prescribed but not obtained
- No medicine prescribed   If this response, jump to 262

261. Why were you not able to get all of the prescribed medicines at the referral facility? (Record the primary reason)
Choose one response
- Medication was out of stock at the referral facility
- Medication was not available at the referral facility
- Medication was too expensive
- Other

262. Did your household have to pay for the referral provider visit?
Payment may include consultation fees for the provider, diagnostic testing, medicines, or transportation to get to/from the facility.
Choose one response
- Yes
- No If this response, jump to 270
- Don't Know   If this response, jump to 270

263. What was the total amount that your household paid at the referral facility (JD)?

264. How much money did your household pay to the referral provider for consultation fees (JD)?

265. How much money did your household pay at the referral facility for diagnostic testing (JD)?

266. How much money did your household pay for medicines at the referral facility (do not include medicines purchased elsewhere; in JD)?

267. Of the medicines you obtained at the referral facility, did you get any on credit?
Choose one response
- Yes
- No If this response, jump to 269

268. How much is still owed (in JD)?

269. How much money did your household pay for transportation to/from the facility (in JD)?

270. Were there any diagnostic tests, treatments, or medicines that were recommended at the referral facility but that you could not afford?
Choose one response
- Yes
- No If this response, jump to 273

271. What was recommended that you could not afford?
Choose all that apply
- Diagnostic tests
- Medication
- Specialist Consultation
- Medical Equipment
- Other

272. What was the total cost of recommended tests/treatments/medicines that were not obtained because of cost (in

JD)?

273. Were any medications prescribed for (name)'s illness that were not obtained at the facilities where care was received?
Choose one response
- Yes
- No If this response, jump to 286

274. Did you attempt to obtain these medications elsewhere?
Choose one response
- Yes
- No If this response, jump to 278

275. Where did you attempt to obtain the prescribed medications?
Choose all that apply
- At another facility (different from where the doctor was seen)
- Private pharmacy
- Syria
- Via a local contact/fixer
- Other

276. Provide description for "other". Leave blank if "other" was not selected in previous question.

277. Were you able to get all of the medications that were prescribed?
Choose one response
- Yes, all of the medications   If this response, jump to 280
- Yes, some of the medications
- No
- Don’t Know   If this response, jump to 286

278. Why were you not able to get all of the medicines that were prescribed? (Record the primary reason)
Choose one response
- Household could not afford the medication
- Did not know where to get the medication
- Medication was out of stock at the facility
- Medication was out of stock at private pharmacy
- Symptoms improved/(name) began to feel better
- Household chose a different treatment
- Household decided the medicines were not needed
- Purchasing medication was not a priority
- Too far to travel to obtain medication
- Other (specify)

279. Provide description for "other". Leave blank if "other" was not selected in previous question.

280. Did your household have to pay for the medications received outside the facilities where you received care?
Choose one response
- Yes
- No If this response, jump to 286

281. How much money did your household pay for medicines outside the facility where you received care (do not include medicines purchased at the care facility; in JD)?

282. Of the medicines you obtained outside the health facilities where care was received, did you get any on credit?
Choose one response
- Yes
- No If this response, jump to 286

283. How much is still owed (in JD)?

284. What was the main reason for not seeking medical care for the health condition? (Record one, primary reason)
Choose one response
- Could not afford provider costs   If this response, jump to 286
- Distance or lack of transportation   If this response, jump to 286
- Could not afford transportation costs   If this response, jump to 286
- Provider's equipment or drugs are inadequate   If this response, jump to 286
- Disliked previous visit(s) (attitude)   If this response, jump to 286
- Disliked previous visit(s) (long waiting time)   If this response, jump to 286
- Disliked previous visit(s) (quality of care)   If this response, jump to 286
- Could not take time off work or had other commitments   If this response, jump to 286
- Didn't know where to go   If this response, jump to 286
- Family decided care should not be sought (no male permission)   If this response, jump to 286
- Security concerns and/or check points   If this response, jump to 286
- Appointment scheduled / still waiting   If this response, jump to 286
- Other (specify)

285. Provide description for "other"

286. SECTION 10: SEXUAL AND REPRODUCTIVE HEALTH

287. Are any women in the household currently pregnant or have they given birth in the past six months?
Choose one response
- No   If this response, jump to 301
- Currently pregnant If this response, jump to 288
- Recently gave birth If this response, jump to 289
- Miscarriage If this response, jump to 301

288. When do you expect the baby to be born?

289. Month of birth
Choose one response
- January
- February
- March
- April
- May
- June
- July
- August
- September
- October
- November
- December

290. Year of Birth
Choose one response
- 2017
- 2018
- 2019

291. In what type of facility did [name] deliver the baby?
Choose one response
- Public hospital
- Private hospital or clinic
- Home
- NGO
- Other

292. What was the type of delivery?
Choose one response
- Vaginal Normal
- Caesarean section
- Don't Know

293. Did [name] have to pay anything herself for this delivery?
Choose one response
- Yes
- No If this response, jump to 295

294. Approximately how much did they pay (in JD)?

295. Did [name] receive any financial support from UNHCR or an NGO to help pay for the delivery costs?
Choose one response
- Yes
- No If this response, jump to 299

296. Was this a payment to the health facility or a cash transfer or reimbursement to [name] directly?
Choose one response
- Health facility If this response, jump to 299
- Individual cash transfer If this response, jump to 298
- Other (specify)

297. Provide description for "other"

298. How much was the transfer/reimbursement (in JD)?

299. Did you know that UNHCR provides support for delivery at hospital?
Choose one response
- Yes
- No If this response, jump to 301

300. Did you know how to access this support?
Choose one response
- Yes
- No

301. SECTION 11: HOUSEHOLD HEALTH CARE ACCESS PERCEPTIONS
I am going to read you five statements about access to medical care. Please tell me if you agree or disagree with each statement.

302. My household is able to get medical care whenever we need it.
Choose one response
- Strongly agree
- Somewhat agree
- Somewhat disagree
- Strongly disagree
- Non applicable or no opinion

303. My household has access to the medical specialists we need.
Choose one response
- Strongly agree
- Somewhat agree
- Somewhat disagree
- Strongly disagree
- Non applicable or no opinion

304. My household can always afford medical care.
Choose one response
- Strongly agree
- Somewhat agree
- Somewhat disagree
- Strongly disagree
- Non applicable or no opinion

305. My household can always afford the medications we need.
Choose one response
- Strongly agree
- Somewhat agree
- Somewhat disagree
- Strongly disagree
- Non applicable or no opinion

306. My household receives enough health information.
Choose one response
- Strongly agree
- Somewhat agree
- Somewhat disagree
- Strongly disagree
- Non applicable or no opinion

307. INTERVIEWER: This is the end of the questionnaire. Please thank the participant for their time.

308. Please write any additional comment about the interview that study coordinators need to know. If possible, please enter comment(s) in English.

309. Did you complete the full interview?
Choose one response

- Yes
- No, but I have arranged to call the respondent back to complete the interview
- No, respondent refused to participate
- No, wrong phone number provided
- No, patient did not meet eligibility criteria

Subform: H3SD_R2HC_MPC_Enrollment_Jordan_NCDSub_v4
65 Questions

==
1. How old is (name) currently (in years)?

2. (Name)'s sex
Choose one response
- Male
- Female

3. What chronic medical conditions has (name) been diagnosed with?
Choose all that apply
- Hypertension
- Diabetes
- Arthritis
- Chronic respiratory disease
- Cardiovascular disease
- Cancer
- Other (specify)

4. Provide description for “other”. Leave blank if "other" was not selected in previous question.

5. When was the last time that (name) saw a doctor for their chronic medical condition while in Jordan?
Choose one response
- Less than 1 month   If this response, jump to 8
- Between 1 month and less than 3 months ago   If this response, jump to 8
- Between 3 months and less than 6 months ago If this response, jump to 8
- Between 6 months and less than 1 year ago   If this response, jump to 8
- More than 1 year ago If this response, jump to 54
- Never needed/sought care in Jordan
- Don’t Know   If this response, jump to 54

6. What was the main reason for not seeking medical care for (name)'s chronic condition? (Record one, Primary reason)
Choose one response
- Could not afford provider costs   If this response, jump to 54
- Distance or lack of transportation   If this response, jump to 54
- Could not afford transportation costs   If this response, jump to 54
- Provider’s equipment or drugs are inadequate   If this response, jump to 54
- Disliked previous visit(s) (attitude)   If this response, jump to 54
- Disliked previous visit(s) (long waiting time)   If this response, jump to 54
- Disliked previous visit(s) (quality of care)   If this response, jump to 54
- Could not take time off work or had other commitments   If this response, jump to 54
- Didn't know where to go   If this response, jump to 54
- Family decided care should not be sought (no male permission)   If this response, jump to 54
- Security concerns and/or check points   If this response, jump to 54
- Appointment scheduled / still waiting   If this response, jump to 54
- Not sick enough to go to health facility   If this response, jump to 54
- Other (specify)

7. Provide description for "other"

8. For the last time (name) needed care for chronic medical conditions in Jordan, where did (name) first go?
Choose one response
- Government primary health care center If this response, jump to 10
- Government comprehensive care center   If this response, jump to 10
- Private Jordanian clinic or doctor If this response, jump to 10
- Islamic charity (free or low cost services to Syrian refugees)   If this response, jump to 10
- NGO or other non-religious charity (free or low cost services to Syrian refugees)   If this response, jump to 10
- Hospital   If this response, jump to 10
- Syrian doctor or clinic   If this response, jump to 10
- Pharmacy If this response, jump to 10
- Shop or Other

9. Provide description for "other"

10. What was the reason for choosing to seek care from that provider? (Record primary reason)
Choose one response
- Free consultation services
- Low cost consultation services
- Closest to place of residence
- No other facility nearby
- Like staff/quality/family doctor
- Free or reduced cost medications provided
- Free or reduced diagnostic tests provided
- Availability of needed services
- Medical emergency
- Short waiting time / easy to get an appointment
- Not sick enough to take to health facility
- Did not know any other providers
- Other (specify)

11. Provide description for other. Leave blank if other was not selected in the previous question.

12. What type of hospital was used?
Choose one response
- Private hospital
- Public hospital
- NGO
- Don't know

13. What type of visit was this?
Choose one response
- Emergency room visit (where people receive urgent care when they are very ill If this response, jump to 20
- Outpatient visit   If this response, jump to 20
- Inpatient admission (when you are given a bed at the hospital and stay overnight or longer)

14. How many nights was the hospital stay?

15. Did UNHCR or any other organization assist in paying for some of the fees?
Choose one response
- Yes, UNHCR
- Yes, other organization

- Yes, both
- No, none
- Don’t Know

16. Did you/your household have to pay for any of the cost yourself?
Choose one response
- Yes
- No If this response, jump to 27
- Don't Know If this response, jump to 27

17. How much did your household pay for the hospital visit (in JD, excluding the amount paid by any organization on your behalf)?

18. Did you pay for the hospital visit on credit?
Choose one response
- Yes
- No If this response, jump to 27

19. How much is still owed (in JD)?

20. Did your household have to pay for the provider visit?
Payment may include consultation fees for the provider, diagnostic testing, medicines, or transportation to get to/from the facility.
Choose one response
- Yes, paid some of the cost
- Yes, paid all of the cost If this response, jump to 22
- No If this response, jump to 27

21. Who paid the other portion of the cost ?

22. What was the total amount that your household paid at the facility? (JD) Do not include amounts that were paid on your behalf or reimbursed.

23. How much money did your household pay to the provider for consultation fees (in JD)?

24. How much money did your household pay for diagnostic testing (JD)?

25. How much money did your household pay for medicines (at the facility, do not include medicines purchased elsewhere; in JD)?

26. How much money did your household pay for transportation to/from the facility (in JD)?

27. Were there any diagnostic tests, treatments, or medicines that were recommended but that you could not afford?
Choose one response
- Yes
- No If this response, jump to 30

28. What was recommended that you could not afford?
Choose all that apply
- Diagnostic tests
- Medication
- Specialist Consultation

- Medical Equipment
- Other

29. What was the total cost of recommended tests/treatments/medicines that were not obtained because of cost (in JD)?

30. Was (name) referred to another facility or provider for care?
Choose one response
- Yes, received referral care / نعم، تقى خدمات اتحوي
- Yes, but did not receive referral care
- No / ا If this response, jump to 50
- Don’t Know If this response, jump to 50

31. What was (name) referred for?
Choose one response
- Lab tests / فحوصات مخبرية
- Diagnostic test / وسائ تشخيص
- Specialist Consultation
- Curative intervention (specific medication, surgery, procedure) / تدخ عاجي - أدوية ، جراحة، عاج مختص
- Other

32. Where was (name) referred?
Choose one response
- Government primary health care center If this response, jump to 34
- Government comprehensive care center If this response, jump to 34
- Private Jordanian clinic or doctor If this response, jump to 34
- Islamic charity (free or low cost services to Syrian refugees) If this response, jump to 34
- NGO or other non-religious charity (free or low cost services to Syrian refugees) If this response, jump to 34
- Syrian doctor or clinic If this response, jump to 34
- Hospital
- Other If this response, jump to 34

33. What type of hospital was used?
Choose one response
- Private hospital
- Public hospital
- NGO
- Don’t know

34. What type of visit was this?
Choose one response
- Emergency room visit (where people receive urgent care when they are very ill) / زيارة غرفة اطوارئ (اماكن اذي يتقى فيه اأشخاص ارعاية اعاجة إذا كانوا شديدي امرض) If this response, jump to 41
- Outpatient visit If this response, jump to 41
- Inpatient admission (when you are given a bed at the hospital and stay overnight or longer)

35. How many nights was the hospital stay?

36. Did UNHCR or any other organization assist in paying for some of the fees?
Choose one response
- Yes, UNHCR
- Yes, other organization

- Yes, both
- No, none
- Don’t Know

37. Did you/your household have to pay for any of the cost yourself?
Choose one response
- Yes
- No   If this response, jump to 47
- Don’t Know   If this response, jump to 47

38. How much did your household pay for the hospital visit (in JD, excluding the amount paid by any organization on your behalf)?

39. Did you pay for the hospital visit on credit?
Choose one response
- Yes
- No   If this response, jump to 47

40. How much is still owed (in JD)?

41. Did your household have to pay for the referral provider visit?
Payment may include consultation fees for the provider, diagnostic testing, medicines, or transportation to get to/from the facility.
Choose one response
- Yes
- No   If this response, jump to 47
- Don’t Know   If this response, jump to 47

42. What was the total amount that your household paid at the referral facility (JD)?

43. How much money did your household pay to the referral provider for consultation fees (in JD)?

44. How much money did your household pay at the referral facility for diagnostic testing (in JD)?

45. How much money did your household pay for medicines referral facility (do not include medicines purchased elsewhere; in JD)?

46. How much money did your household pay for transportation to/from the facility (in JD)?

47. Were there any diagnostic tests, treatments, or medicines that were recommended at the referral facility but that you could not afford?
Choose one response
- Yes
- No   If this response, jump to 50

48. What was recommended that you could not afford?
Choose all that apply
- Diagnostic tests
- Medication
- Specialist Consultation
- Medical Equipment / امعدات/أجهزة اطبية
- Other

49. What was the total cost of recommended tests/treatments/medicines that were not obtained because of cost (in JD)?

50. How many times has (name) visited a General Practitioner (GP) in the last 6 months?

51. How many times has (name) seen another doctor/specialist (not GP) in the last 6 months?

52. How many times has (name) consulted a pharmacist in the last 6 months?

53. How many visits to a hospital for chronic medical conditions has (name) had in the last 6 months?

54. Has (name) been prescribed, by a doctor/pharmacist, medicines for their chronic condition(s) (in Jordan or in Syria)?
Choose one response
- Yes, by a doctor in Syria
- Yes, by a pharmacist in Syria
- Yes, by a doctor in Jordan
- Yes, by a pharmacist in Jordan
- No   If this response, jump to 65

55. How many medications is (name) currently prescribed for chronic conditions?

56. In the past month, how often has (name) taken the medications prescribed for chronic conditions?
Choose one response
- Always
- Most of the time (>50% of doses taken as prescribed)
- Some of the time (<50% of doses taken as prescribed)
- Never (did not take medication in the past month)

57. Does the household face difficulties obtaining medication prescribed for (name)?
Choose one response
- Yes, very often
- Yes, sometimes
- Never   If this response, jump to 60

58. What difficulties did you face in obtaining medication? (Record primary difficulty)
Choose all that apply
- Medication was out of stock at the facility
- Household could not afford the medication
- Did not know where to get the medication
- Difficulty finding a pharmacy with my medication
- Too far to travel to obtain medication
- Did not like what was available
- Other (specify)

59. Provide description for "other". Leave blank if "other" was not selected in previous question.

60. Where does (name) most often obtain medication?
Choose one response
- At the facility where the doctor was seen   If this response, jump to 62
- At another facility (different from where the doctor was seen) If this response, jump to 62

- Private pharmacy If this response, jump to 62
- Syria If this response, jump to 62
- Via a local contact/fixer If this response, jump to 62
- Other (specify)

61. Provide description for "other"

62. On average, how much does the household pay for (name)’s medications per month (in JD)?
If medication received in longer supply, calculate average monthly price (i.e. for 3-month supply divide cost by 3).

63. Have you received any medicines for (name)'s condition on credit?
Choose one response
- Yes
- No If this response, jump to 65

64. How much is still owed (in JD)?

65. End of chronic condition questions for this individual
