# Supplementary material for: Multi-purpose cash transfers and health among vulnerable Syrian refugees in Jordan: A prospective cohort study
Source: PLOS Glob Public Health. 2022 Nov 2;2(11):e0001227. doi: 10.1371/journal.pgph.0001227 (PMC10021566; doi:10.1371/journal.pgph.0001227)
Supplement: S1 Table — Baseline and endline descriptive analyses of care-seeking outcomes by group. (PDF) [file pgph.0001227.s004.pdf]

Health Care-Seeking and Medicines for Household Member Illness at Baseline and Endline

		BASELINE			ENDLINE		
		**MPC HHs (N=429)** % (95% CI)	**Control HHs (N=448)** % (95% CI)	P value	**MPC HHs (N=411)** % (95% CI)	**Control HHs (N=391)** % (95% CI)	P value
**Most Recent Childhood Illness**							
Sought and received medical care		77.0% (70.5,83.5%)	87.4% (83.3,91.4%)	**0.014**	82.9% (77.8,87.9%)	84.2% (80.0,88.4%)	0.254
Able to obtain prescribed medications		96.6% (93.3,99.9%)	98.1% (96.2,100.0%)	0.411	100% --	98.8% (97.4,100.2%)	0.143
Outpatient visit		71.1% (63.1,79.1%)	78.1% (72.7,83.5%)		93.3% (89.6,97.0%)	94.3% (91.4,97.2%)	
Emergency room visit		25.8% (18.1,33.5%)	19.7% (14.5,24.9%)	0.337	3.9% (1.0,6.8%)	4.1% (1.6,6.6%)	0.710
Hospital admission		3.1% (0.1,6.2%)	2.2% (0.3,4.1%)		2.8% (0.4,5.2%)	1.6% (0.0,3.2%)	
**Care Facility Sector**	Public	22.8% (15.0,30.6%)	23.8% (17.8,29.7%)		26.7% (19.1,34.2%)	24.7% (18.3,31.1%)	
	Private	62.3% (53.2,71.3%)	68.3% (61.8,74.8%)	0.147	57.8% (49.3,66.2%)	66.3% (59.3,73.3%)	0.149
	Charity	14.9% (8.3,21.6%)	7.9% (4.2,11.7%)		15.6% (9.4,21.7%)	9.0% (4.7,13.2%)	
Medical care not sought because of cost		92.1% (83.1,101.1%)	87.1% (74.6,99.6%)	0.493	100% --	91.3% (82.8,99.8%)	*0.074*
All needed care not received due to cost		18.0% (11.2,24.7%)	11.0% (6.9,15.1%)	*0.063*	6.7% (3.0,10.4%)	6.5% (3.4,9.6%)	0.943
**Most Recent Adult Acute Illness**							
Sought and received medical care		68.3% (59.9,76.8%)	73.5% (66.0,81.0%)	**0.031**	63.9% (58.5,69.4%)	68.1% (63.0,73.2%)	*0.091*
Able to obtain prescribed medications		94.7% (89.5,99.9%)	94.7% (90.1,99.3%)	0.997	98.9% (97.4,100.4%)	99.0% (97.7,100.4%)	0.898
Outpatient visit		72.0% (62.0,81.9%)	89.0% (82.8,95.2%)		97.4% (95.1,99.7%)	94.1% (90.9,97.2%)	
Emergency room visit		20.7% (11.8,29.7%)	11.0% (4.8,17.2%)	**0.003**	2.6% (0.3,4.9%)	5.9% (2.8,9.1%)	*0.097*
Hospital admission		7.3% (1.6,13.1%)	0.0% --		0.0% --	0.0% --	
**Care Facility Sector**	Public	27.4% (16.9,37.9%)	16.3% (8.3,24.2%)		27.7% (19.3,36.1%)	22.5% (15.4,29.5%)	
	Private	54.8% (43.1,66.5%)	74.4% (65.0,83.8%)	**0.034**	50.0% (40.6,59.4%)	61.6% (53.4,69.8%)	0.175
	Charity	17.8% (8.8,26.8%)	9.3% (3.0,15.6%)		22.3% (14.5,30.2%)	15.9% (9.8,22.1%)	
Medical care not sought because of cost		84.2% (72.1,96.4%)	80.6% (67.0,94.1%)	0.680	94.5% (90.1,98.8%)	96.1% (92.3,99.9%)	0.578
All needed care not received due to cost		24.4% (14.9,33.9%)	18.0% (10.3,25.7%)	0.291	6.7% (3.2,10.3%)	11.1% (6.9,15.3%)	0.127
**Adult Chronic Illness**							
Sought and received medical care		93.0% (90.8,95.1%)	92.0% (89.0,95.0%)	0.606	96.3% (94.5,98.0%)	97.2% (95.1,99.2%)	0.543
Ever faced difficulties obtaining medication		68.6% (64.6,72.7%)	70.2% (65.0,75.4%)	0.642	57.8% (53.1,62.5%)	59.8% (53.6,66.1%)	0.604
General practitioner visit(s)		63.2% (58.6,67.8%)	59.2% (53.2,65.2%)	0.289	65.8% (61.0,70.7%)	65.1% (58.4,71.9%)	0.864
Specialist visit(s)		56.1% (51.4,60.9%)	62.0% (56.1,67.9%)	0.131	53.4% (48.3,58.6%)	52.8% (45.8,59.9%)	0.891
Hospital visit(s)		42.3% (37.6,46.9%)	40.1% (34.1,46.0%)	0.571	34.7% (29.8,39.6%)	26.0% (19.8,32.2%)	**0.035**
**Care Facility Sector**	Public	22.0% (18.0,25.9%)	29.6% (24.0,35.2%)		19.4% (15.3,23.5%)	20.6% (14.8,26.5%)	
	Private	44.6% (39.9,49.4%)	40.5% (34.4,46.5%)	0.084	41.6% (36.4,46.7%)	46.0% (38.9,53.2%)	0.416
	Charity	33.4% (28.9,37.9%)	30.0% (24.3,35.6%)		39.0% (34.0,44.1%)	33.3% (26.6,40.1%)	
Medical care not sought because of cost		60.0% (36.5,83.5%)	70.6% (46.4,94.7%)	0.501	62.5% (19.2,105.8%)	100% --	0.301
All needed care not received due to cost		32.5% (28.1,36.9%)	26.1% (20.8,31.5%)	*0.076*	21.9% (17.6,26.1%)	22.4% (16.6,28.3%)	0.872
Cannot afford medication		61.6% (57.4,65.8%)	61.0% (55.7,66.4%)	0.872	42.1% (37.6,46.7%)	45.9% (39.7,52.2%)	0.333
