# Supplementary material for: Multi-purpose cash transfers and health among vulnerable Syrian refugees in Jordan: A prospective cohort study
Source: PLOS Glob Public Health. 2022 Nov 2;2(11):e0001227. doi: 10.1371/journal.pgph.0001227 (PMC10021566; doi:10.1371/journal.pgph.0001227)
Supplement: S2 Table — Baseline and endline descriptive analyses of health expenditure outcomes by group. (PDF) [file pgph.0001227.s005.pdf]

Health Expenditures for Most Recent Child, Adult Acute, and Adult Chronic Illness Care and in the Preceding Month (USD [a]) at Baseline and Endline

	BASELINE					ENDLINE				
	MPC HHs (N=429)		Control HHs (N=448)			MPC HHs (N=411)		Control HHs (N=391)		
	Mdn	Mean (95% CI)	Mdn	Mean (95% CI)	P value	Mdn	Mean (95% CI)	Mdn	Mean (95% CI)	P value
**Most Recent Childhood Illness**										
**Health Facility Payments for OP Care [b]**		*n=123*		*n=223*			*n=174*		*n=241*	
Any payment for outpatient care at facility	--	72.4% (64.3,80.4%)	--	78.0% (72.6,83.5%)	0.237	--	81.6% (75.8,87.4%)	--	89.2% (85.3,93.2%)	**0.028**
Total paid at facility for visit (all HHs)	10	17.2 (12.7,21.7)	10	22.5 (14.4,30.7)	0.362	10	24.9 (13.9,35.9)	14	16.6 (14.1,19.0)	*0.090*
**Medication Costs at Pharmacy/Elsewhere**		*n=123*		*n=223*			*n=174*		*n=242*	
Any payment for medication outside facility	--	34.1% (25.6,42.6%)	--	41.7% (35.2,48.2%)	0.168	--	34.5% (27.4,41.6%)	--	34.7% (28.7,40.8%)	0.962
Total paid for medication (all HHs)	0	7.1 (3.5,10.6)	0	7.4 (5.8, 9.1)	0.844	0	5.4 (3.8, 7.0)	0	6.7 (5.2, 8.3)	0.260
**Total Amount Paid for Illness [c]**		*n=123*		*n=223*			*n=174*		*n=241*	
Any expense for most recent illness	--	69.1% (60.8,77.4%)	--	72.2% (66.3,78.1%)	0.544	--	71.3% (64.5,78.1%)	--	81.3% (76.4,86.3%)	**0.016**
Total cost for most recent illness (all HHs)	13	20.8 (14.9,26.7)	14	21.4 (17.1,25.7)	0.883	14	22.6 (14.2,31.1)	14	21.2 (18.1,24.3)	0.725
**Most Recent Acute Adult Illness**										
**Health Facility Payments for OP Care [b]**		*n=76*		*n=100*			*n=192*		*n=216*	
Any payment for outpatient care at facility	--	64.5% (53.5,75.5%)	--	76.0% (67.5,84.5%)	*0.095*	--	75.5% (69.4,81.7%)	--	83.8% (78.8,88.7%)	**0.037**
Total paid at facility for visit (all HHs)	9	21.5 (8.9,34.2)	14	29.4 (20.9,37.9)	0.287	9	16.3 (11.6,21.1)	14	24.6 (16.7,32.6)	*0.088*
**Medication Costs at Pharmacy/Elsewhere**		*n=76*		*n=100*			*n=193*		*n=219*	
Any payment for medication outside facility	--	32.9% (22.1,43.7%)	--	44.0% (34.1,53.9%)	0.135	--	15.0% (9.9,20.1%)	--	25.6% (19.7,31.4%)	**0.008**
Total paid for medication (all HHs)	0	7.0 (3.8,10.2)	0	10.4 (6.0,14.9)	0.232	0	3.7 (1.8, 5.6)	0	5.6 (3.7, 7.4)	0.174
**Total Amount Paid for Illness [c]**		*n=76*		*n=100*			*n=192*		*n=216*	
Any expense for most recent illness	--	65.8% (54.9,76.7%)	--	75.0% (66.4,83.6%)	0.182	--	67.7% (61.0,74.4%)	--	81.0% (75.7,86.3%)	**0.002**
Total cost for most recent illness (all HHs)	14	18.6 (13.0,24.1)	18	32.3 (23.6,41.0)	**0.015**	7	16.6 (12.0,21.1)	14	28.0 (19.9,36.1)	**0.019**
**Most Recent Adult Chronic Illness Visit**										
**Health Facility Payments for OP Care [b]**		*n=428*		*n=258*			*n=355*		*n=193*	
Any payment for outpatient care at facility	--	59.6% (54.9,64.2%)	--	63.6% (57.7,69.5%)	0.300	--	63.7% (58.6,68.7%)	--	62.7% (55.8,69.6%)	0.822
Total paid at facility for visit (all HHs)	10	34.4 (27.3,41.6)	9	27.5 (20.9,34.1)	0.194	7	25.8 (19.5,32.1)	7	43.0 (13.9,72.2)	0.144
**Average Monthly Medication Costs**		*n=526*		*n=326*			*n=458*		*n=246*	
Any regular (monthly) medication costs	--	64.4% (60.3,68.6%)	--	63.5% (58.2,68.8%)	0.778	--	50.9% (46.3,55.5%)	--	55.7% (49.4,61.9%)	0.222
Average monthly medication costs (all cases)	14	27.2 (23.7,30.6)	11	22.1 (18.8,25.3)	**0.048**	4	20.2 (17.3,23.2)	7	16.9 (13.8,20.0)	0.155
**Routine Spending on Health**		*n=429*		*n=448*			*n=411*		*n=391*	
Health Expenditures (past month) [d]	38	73.8 (60.7,86.8)	28	53.1 (43.5,62.7)	**0.012**	42	74.4 (50.7,98.0)	28	48.1 (41.3,54.8)	**0.039**
Sold assets to pay for health (past 3 months)	--	6.8% (4.4,9.1%)	--	8.7% (6.1,11.3%)	0.282	--	7.5% (5.0,10.1%)	--	10.5% (7.4,13.5%)	0.145
Borrowed to pay for health (past 3 months)	--	39.9% (35.2,44.5%)	--	33.7% (29.3,38.1%)	*0.059*	--	35.0% (30.4,39.7%)	--	35.8% (31.0,40.6%)	0.820

HH = households; Mdn = median; OP = outpatient. [a] reported in USD; exchange rate: 1 JOD = 1.41 USD; [b] includes consultation fees, diagnostic testing and medications obtained at health facility during the initial visit to health facility, hospital outpatient department, or emergency room (without overnight stay); [c] includes health facility payments for outpatient care and medications purchased at pharmacies outside health facilities (does not include referrals, in-patient care and transportation); [d] at facilities and for medication
