# Supplementary material for: Multi-purpose cash transfers and health among vulnerable Syrian refugees in Jordan: A prospective cohort study
Source: PLOS Glob Public Health. 2022 Nov 2;2(11):e0001227. doi: 10.1371/journal.pgph.0001227 (PMC10021566; doi:10.1371/journal.pgph.0001227)
Supplement: Attachment — Submitted filename: Jordan MPC - Response to Reviewers.docx [file pgph.0001227.s006.docx]

**Response to Reviewer Comments**

**Journal Requirements:**

1. Please include a complete copy of PLOS’ questionnaire on inclusivity in global research in your revised manuscript. Our policy for research in this area aims to improve transparency in the reporting of research performed outside of researchers’ own country or community. The policy applies to researchers who have travelled to a different country to conduct research, research with Indigenous populations or their lands, and research on cultural artefacts. The questionnaire can also be requested at the journal’s discretion for any other submissions, even if these conditions are not met.  Please find more information on the policy and a link to download a blank copy of the questionnaire here: [https://journals.plos.org/globalpublichealth/s/best-practices-in-research-reporting](https://nam02.safelinks.protection.outlook.com/?url=https%3A%2F%2Fjournals.plos.org%2Fglobalpublichealth%2Fs%2Fbest-practices-in-research-reporting&data=04%7C01%7Celyles2%40jhu.edu%7Cc800dc8db29e40494a8c08da13239378%7C9fa4f438b1e6473b803f86f8aedf0dec%7C0%7C0%7C637843340760610708%7CUnknown%7CTWFpbGZsb3d8eyJWIjoiMC4wLjAwMDAiLCJQIjoiV2luMzIiLCJBTiI6Ik1haWwiLCJXVCI6Mn0%3D%7C3000&sdata=ckF%2BAd4a7Tm1n8S1CICcnTsdB%2FUJHIyOhBtdlD1X4WM%3D&reserved=0). Please upload a completed version of your questionnaire as Supporting Information when you resubmit your manuscript.

Authors’ Response: The questionnaire was completed and is also referenced in the text as supporting information.

1. Please add a 150- to 200-word non-technical author summary to your article file. The author summary should come after the abstract but before the introduction.

Authors’ Response: An author summary has been added after the abstract as requested.

**Reviewer #1:**

The paper provides evidence from the MPC in Jordan and its relation with the health care need, utilization and expenditure of refugees. The study's main strength is the data collection that addresses to the aim of the study. Even if I find the findings of the study very interesting I have some reservations about the institutional set up during the study period. Especially potential uncertainty generated by the institutional set up which makes it difficult to interpret the analysis results. My main concerns are around i) GOJ's health policy change in March 2018 and March 2019, ii) UNHCR's revision of the MPC eligibility criteria (lines 179-180) and resulting change in the eligibility status of some families, iii) WFP's coverage change during the study period. The authors do explain these limitations thoroughly in the Discussion session. I believe the study will improve if these are brought up earlier in the Introduction and the institutional set up and their relation to the study findings will be better understood by the readers. Especially, the authors should put more emphasis on whether we should expect any differential effect of these changes on the treatment and control groups.

Authors’ Response: The introduction was revised to make more explicit the three main concerns noted by reviewer #1 earlier in the article. We did not have particular expectations about outcomes, mostly because we were uncertain if the transfers would have measurable impacts on health due to both 1) the diversity of health needs across households and their members, and 2) the size of the transfer compared to the extent of unmet needs and household priorities for other types of expenditures (e.g., food, rent).

Additionally here are couple of points that needs clarification:

Table 2: The treated move to apartments by the endline. Why would that happen? Are there any concurrent policies about the housing of the refugees? Does this have to do with the MPC? How does this affect the refugees health? Do they also change neighborhood?

Authors’ Response: We are not aware of any substantial changes to shelter programming or policies during the study period. Increases in the proportion of households living in apartments/single family homes increased in both the intervention and control group over the course of the study period. While the magnitude of increase was greater in the MPC group (8.6%) compared to the control group (4.5%), at endline the proportion of households in each group residing in apartments/single family homes was very similar in the two groups (89.3% vs 90%). It is possible that the intervention group used transfers to pay for rent which could have enable them to move to apartments/single family homes, however, this was not a topic of investigation in this study. Similarly, we did not examine the relationship between dwelling type and the health outcome measures but established evidence suggests that poor living conditions (e.g., lack of access to water and sanitation, crowding) are associated with poorer health status. Finally, information on precise geographic location of residence (i.e., neighborhood) was not collected outside of the initial sampling list from UNHCR so we cannot determine if participants also changed neighborhoods nor what the implications on health may be for these 21 households.

Table S3: I see that the outpatient visits increase for control and treatment by the endline? This may be due to the GOJ policy change in March 2019 or a simultaneous increase in the health care workers supply or health infrastructure buildup. Needs to be elaborated.

Authors’ Response: We are not aware of any substantial increases in the number of health workers or large scale changes in health infrastructure that occurred during the study period that would have substantially improved supply/access to services at a national level. Changes to GoJ policy, including in 2019, are summarized in the introduction, however, it is difficult to attribute observed differences to a specific factor such as policy change with the given study design. Furthermore, data was not collected on the awareness of policy change among refugees and adoption of policy change at health facilities (which was perceived as uneven) which likely be needed to do a policy analysis. Difference in change in care seeking rates was statistically significant for only one of three groups (i.e. child, adult acute, adult chronic) and the direction of change was no always consistent, making it seem unlikely that widespread policy and health systems changes are significant drivers of increased demand/reduced barriers for care.

Study Sample: What are the characteristics of the households that dropped from the program? These should be provided in a summary table.

Authors’ Response: Tables presenting baseline characteristics of participants lost to follow-up have been added to supporting file 1 along with supplemental sample methods.

MPC program eligibility: How do the authors interpret revisions in the MPC and their relation to the outcome variables? Do the program rules generate any incentives for the manipulation of health status or household composition for the applicants or beneficiaries?

Authors’ Response: The Vulnerability Assessment Framework (VAF) used to determine eligibility for MPC receipt incorporates a substantial number of multi-sectoral indicators and models/formulae for establishing vulnerability scores. The nature of this targeting approach is intentional for mitigating the likelihood of manipulation of any single indicator or set of indicators substantially altering eligibility for MPC, thus, limiting the incentive for households to manipulate information provided during household visits to this end. Text was added to the manuscript’s discussion of the VAF targeting approach to make this more explicit.

Method: The inability to check the parallel trends assumption is a big drawback in this setting. There is reason to believe that the MPC recipients and the control follow different trends. One potential area of concern is the duration of cash transfers and how long the MPC recipients were supported or whether the control group households were previously supported or not. For this reason if the dataset includes a variable to control for these points, it will be nice to introduce it in the text.

Authors’ Response: We agree with the reviewer’s point; however, aside from MPC receipt during the study, we do not have the necessary data to control for these factors.

Minor comments: the references need to be put in order. Reference 32 (mentioned in line 558) is not a study about Turkey.

Authors’ Response: Reference numbering was checked and all references were updated/corrected where needed.

**Reviewer #2**

This is an interesting study addressing the relevant topic of health financing in humanitarian settings and contributing to generating evidence on the modality of multi-cash vouchers. While this study clearly adds to the evidence base of this topic, it can benefit from a review to improve the quality of the manuscript especially in terms of results' structure and discussion. Below are some points to be addressed by authors.

Introduction: clear introduction and good framing of the study background especially in relation with the Jordanian context and introducing cash assistance. However, the comparison between cash assistance and in-kind assistance (of households/individuals?) might not apply to health as it does to other sectors. Authors can compare this modality to in-kind assistance of health centres or other health financing approaches and/or highlight the gaps in the literature about this comparison (here or in the discussion).

Authors’ Response: Text was added to the introduction highlighting the limited evidence comparing cash to in-kind health assistance/health financing approaches as suggested.

Methods:
- the 'risk of cross-over' justifying the selection of the target of population is not clear to readers and a reflection on this decision on the discussion could help generate inferences about the impact of MPC in other households.

Authors’ Response: Text was added to clarify what is meant by the ‘risk of cross-over’.

- the selection of the most conservative proportion of 50% as well as the minimum difference level in the sample size calculation needs to be justified in relation to what expected based on similar study (Lebanon's study or perhaps (in the time of study design) other studies).

Authors’ Response: Additional text was added clarifying that the most conservative proportion was selected because of an absence of comparable studies at the time of study design.

- table 1 (or at least the section on the analyzed sample) could fit better in the results section as it might lead to confusion among readers in its current position.

Authors’ Response: Table 1 was moved to the beginning of the results section as suggested.

- please add an explicit list of eligibility criteria and consequently explain whether the adult household member (replacing the eligible participant) meets those criteria. A short description of the percentage of those participants and their characteristics is essential if available.

Authors’ Response: Text was added explicitly listing eligibility criteria (which was based at the household level) in addition to clarification that “replacement” adult household members serving as respondents were from the sampled household and, thus, necessarily met eligibility criteria. Information regarding respondents’ position in relation to the principal applicant on the sampled UNHCR case was added to the results section of the manuscript as suggested.

- please clarify the ethical arrangements to use the phone numbers of UNHCR-registered refugees and how the team handled the use of these identifiers.

Authors’ Response: Detailed information regarding ethical implementation of UNHCR-provided refugee lists and phone numbers was added to the “Study Implementation” sub-section of the Methods section.

Results:
- this is a very rich section, but could benefit from sub-headings to support the reader throughout the data presentation. Given the lack of variable definition section in the methods, some variables remain unclear (e.g. how is 'household size' is defined especially for across different residence type; 'in-kind assistance', etc).

Authors’ Response: Sub-headings are currently included in the results section (study population characteristics, health service utilization, and health expenditures). As these sub-sections include mixed discussion across more granular topics, it is difficult to add reliable further sub-headings; however, we are happy to add them if the reviewer has specific suggestions on this. Suggested definitions were added to the methods section and table where relevant; additional definitions can be added in a supplemental section if needed, however, in many cases this level of detail was not perceived as necessary and our preference was to limit the length of the manuscript.

- there is a discrepancy between the findings about household economy in table 3 and those in the corresponding text. please review them.

Authors’ Response: Clarification was added to the text and Table 3 making more explicit references to any regular cash transfers/assistance; however, if there is another discrepancy to which the reviewer is referring, it would be helpful to clarify what it is in order to correct it.

- please briefly explain the reasons behind the selection of confounders for the adjusted estimates, ie in the statistical analysis (either here or in the methods section)

Authors’ Response: Explanation of selection of model covariates *a priori* to include characteristics known or suspected to be associated with intervention receipt and outcomes of interest was added to the corresponding portion of the methods section.

- please explain why the visits to pharmacies were omitted from the sources of healthcare and how this major gap in the available data is addressed

Authors’ Response: Visits to pharmacies accounted for ~10% or fewer visits for all care seeking types (child, adult acute, adult chronic). Because care seeking at a pharmacy (as opposed to a licensed clinician) was not consider ideal and care seeking outside health facilities was relatively low, we did not focus on this outcome [and many of the follow-up questions also did not apply].

- in the presentation and interpretation of findings, could you comment whether a statistically significant DiD could have been reached in case of higher sample size (ie increased power of the study), and whether inferences can (or not) be made especially when the adjusted estimates for the comparison groups are in different direction and statistically significant for instance.

Authors’ Response: Comments were added to the limitations section noting the likely impact of sub-sample analyses (i.e., restricted denominators/small sample sizes) on statistical power and implications for making inferences as suggested. We hope this adequately addresses the reviewer’s comment—it was unclear what type of response was needed and we are open to making further revisions.

- please avoid statements like 'not statistically smaller' (better use simply: not statistically different?) and 'somewhat' protective in describing the evidence emerging from the study.

Authors’ Response: Revisions to language were made as suggested.

Discussion:
It is recommended to write a concise discussion with less results (ie avoiding the repetition of detailed results and perhaps summary statements of the key findings for each outcome) and more comparison with the wider literature as well as the global health implications of the study and perhaps future research. here are some specific points to addressed:

Authors’ Response: Given the large number of outcomes across different areas of health (e.g., child, adult acute, and adult chronic illness) and the varied nature of results, the discussion was intentionally framed in this way to tie findings together, presenting themes in the findings and discussing each in the context of the broader literature and/or implications for program/policy decisions in the future. We removed repetitive reporting of numeric results in the discussion but kept narrative points as we believe mentioning key findings/results through these statements integrating related outcomes is necessary to contextualize and justify their related discussion points. We have addressed the specific points listed below by the reviewer and provided a response to each.

- the first sentence is not clear - could you please explain the difference between frequency of needing care and care-seeking rates. please avoid introducing new variables and results in the discussion.

Authors’ Response: Text was added to clarify care-seeking rates as actualized seeking of care whereas frequency of needing care refers to needing, though not necessarily seeking care.

- In case of re-writing of the discussion section based on the previous recommendation, please use the space to discuss (with more details using a comparative lens for the current study) the mixed results from other studies in Jordan and Lebanon)

Authors’ Response: We have revised the discussion to address the other specific points mentioned, however, we did not address this point about a detailed comparison of the current study to findings from Lebanon. Upon acceptance of this paper, we plan to finalize and submit an additional manuscript that will include the comparative analysis of the Jordan and Lebanon studies as well as an analysis data from Turkey that also looks at multipurpose cash transfers and their impact on heath.

- the following statement "This may suggest that MPC effects on increased health expenditure were realized closer to when transfers were first received and then were sustained (and thus did not change during the study period)" is unclear and introduced confusion as transfer timing was not discussed before in the manuscript. Please explain or drop.

Authors’ Response: This sentence has been removed.

- the discussion referred to very interesting evidence from the literature about how to improve the use of cash assistance, but very briefly. Examples include the need to have higher transfer amounts (line 573) and the limited positive impact of conditional transfers on helath outcomes (line 581). Please elaborate more your discussion (with perhaps a quick reflection on the quality of evidence / studies especially in the case of the second example).

Authors’ Response: Discussion of the referenced studies was expanded as suggested. However, it is important to note - as mentioned in the discussion – that in the case of this study, expenditures exceeded the minimum expenditure basket (MEB) amount, thus it is hard to make a justification for larger transfer size (in this case transfers comprised about half of household budgets) – especially given limited resource ability. We do not think the quality of available of evidence is a particular concern – but it is clear from available evidence that when given choice beneficiaries spend differently, but most often on basic needs (food, rent, health) but there is no ‘right way’ to use multipurpose cash when meeting basic needs because household needs/priorities vary.
