# Supplementary material for: Multi-purpose cash transfers and health among vulnerable Syrian refugees in Jordan: A prospective cohort study
Source: PLOS Glob Public Health. 2022 Nov 2;2(11):e0001227. doi: 10.1371/journal.pgph.0001227 (PMC10021566; doi:10.1371/journal.pgph.0001227)
Supplement: Attachment — Submitted filename: Jordan MPC - Response to Reviewers.docx [file pgph.0001227.s007.docx]

**Response to Reviewer Comments**

1. Please ensure that the funders and grant numbers match between the Financial Disclosure field and the Funding Information tab in your submission form. Note that the funders must be provided in the same order in both places as well.

Authors’ response: Funders and grant numbers have been checked and corrected where needed to meet this requirement.

2. Please revisit reviewer #1's comments and the related revisions and response. (Reviewer #1 comment: “The authors addressed the limitations raised by the reviewers in the Letter. However, I do not see that these changes were made in the main text. In the Letter, the authors mention that "The introduction was revised to make more explicit the three main concerns noted by reviewer #1 earlier in the article." however I do not see these revisions taking place. In this way, the revised manuscript does not address to my main concerns regarding the methodology which leaves very limited scope to improve/distinguish this current work in comparison to what the authors already presented in a previous work, cited below. Lyles E, Arhem J, K houry G, Trujillo A, Spiegel P, Burton A, et al. Multi-purpose cash transfers and health among vulnerable Syrian refugees in Lebanon: A prospective cohort study. BMC Public Health. 2021;21: 1176. doi: 10.1186/s12889-021-11196-8.”

Authors’ response: Following revisions to highlight any of the initial concerns not previously noted in the introduction, we now believe the manuscript now addresses Reviewer 1’s comment to bringing up the three listed concerns earlier in the introduction:

Concern 1) “GOJ's health policy change in March 2018 and March 2019” see manuscript lines 76-85 (page 4)

Concern 2) “UNHCR's revision of the MPC eligibility criteria and resulting change in the eligibility status of some families” see manuscript lines 110-116 (page 6)

Concern 3) “WFP's coverage change during the study period” see manuscript lines 126-138 (pages 6-7)

Our response in the previous revision’s letter regarding greater emphasis on whether any differential effect of these changes on the treatment and control groups should be expected has also now been added to the introduction text (lines 171-174, page 8) per Reviewer 1’s suggestion.

3. Please elaborate in the introduction how the submitted study adds to your related works [26] mentioned by reviewer #1.

Authors’ response: Text was added to initial mention of the parallel study in Lebanon to note that the studies used the same design to assess comparable intervention and in turn, expand the evidence base for multiple settings (lines 184-188, page 9).
